# Protection against Prenatal Alcohol-Induced Damage

**DOI:** 10.1371/journal.pmed.0030196

**Published:** 2006-04-18

**Authors:** Catherine Y Spong

## Abstract

Spong discusses a new animal study that showed that nicotinamide had a protective effect against prenatal alcohol-induced brain damage.

Fetal alcohol syndrome (FAS) is the most common, nongenetic cause of mental retardation, and has a significant national health impact [ 1]. Meeting the criteria for this condition requires the identification of specific features, involving craniofacial dysmorphology (particularly midfacial anomalies), growth retardation, and deficits in brain function. Most children with alcohol-induced prenatal deficits do not have this full syndrome, but, instead, they fall under the umbrella term of fetal alcohol spectrum disorder (FASD). These disorders result from prenatal alcohol exposure, and the sequelae include specific neurodevelopmental and behavioral disorders.

Recent reports suggest that fetal alcohol spectrum disorders affect one out of 100 children born in the United States [ 2]. There have been numerous public health approaches to alerting women to the dangers of drinking during pregnancy—including warning labels appearing on alcoholic beverages in 1989 ( Box 1), and a US Surgeon General warning issued in 1981 (updated and reissued in 2005) urging women who are pregnant or who may become pregnant to abstain from alcohol ( http://www.hhs.gov/surgeongeneral/pressreleases/sg02222005.html). Yet despite these warnings, alcohol use during pregnancy is common, with up to 50% of women of childbearing age consuming alcohol, 15%–20% acknowledging continuing to drink when pregnant, and one in 25 pregnant women reporting binge drinking [ 3, 4].

## Can We Limit the Damage to the Fetus?

It is critically important to understand the role of alcohol's effects during pregnancy for two main reasons. First, such knowledge will help to bolster public health warnings about drinking during pregnancy. Second, a better understanding of how alcohol causes fetal damage will help in developing preventive interventions to protect the fetus in situations where it is exposed to alcohol.

Researchers have evaluated a number of therapies for the prevention of alcohol-induced damage in model systems. These therapies include neuroprotective peptides [ 5–7] and antioxidants targeting the prevention of ethanol-induced apoptosis [ 8, 9]. And now Ieraci and Herrera report in *PLoS Medicine* that nicotinamide, a drug that has been used in humans to treat autoimmune diseases such as diabetes and bullous pemphigoid, protects against ethanol-induced apoptotic neurodegeneration in the developing mouse brain [ 10]. Their study provides a substantial contribution toward finding a treatment to prevent alcohol-induced damage.

Ieraci and Herrera treated seven-day-old mice with 20% ethanol. The animals were also treated with nicotinamide at varying doses and time points; in each litter, animals were equally distributed into the different treatment groups. Cytochrome-c release (which is released from mitochondria during oxygen–glucose deprivation) was evaluated using Western Blot in forebrain tissue. Blood and brain samples were tested at specific time points to quantify ethanol levels. To assess for neurodegeneration, Fluoro-Jade-B staining was performed histologically on perfused and sectioned brain slices. Behavioral tests were performed to evaluate for alcohol-induced impairments. After weaning, offspring were tested using the open field maze to assess spontaneous motor activity, the elevated plus maze to evaluate anxiety and activity, and the fear conditioning test to assess hippocampal learning and memory.

In their study, the authors evaluated the role of nicotinamide during ethanol exposure, focusing on the etiology of alcohol-induced microcephaly. Their work builds upon the known role of caspase-3 activation in the forebrain, which triggers apoptotic neuronal death after ethanol exposure. The authors report that nicotinamide administration reduced the activation of caspase-3 in the forebrain. It also prevented ethanol-induced neuronal death in specific brain regions and long-term behavioral disturbances in activity and learning. Ethanol-treated mice usually show increased activity, and nicotinamide prevented this alcohol-induced increase in activity—as assessed by the number of times an animal crossed a horizontal line and reared onto two legs (i.e., assessment of both horizontal and vertical activity). In addition, nicotinamide prevented ethanol learning impairment in the fear-conditioning paradigm, where animals associate a context with a physical shock.

## Strengths and Limitations of the Approach

As with all animal models, the limitations of Ieraci and Herrera's work lie in translating the findings to humans and in using the findings to identify and optimize treatment strategies. Clearly, significant work is needed to take the study findings further.

A simpler method for the prevention of alcohol-induced damage in pregnancy is obviously the cessation of alcohol consumption. However, given the widespread and apparently increasing fetal exposure to alcohol, it is unlikely that such cessation will occur in the ensuing decades. Therefore, research such as that by Ieraci and Herrera is essential to enhance our understanding and to develop therapeutic interventions.

Ieraci and Herrera have elegantly evaluated one such intervention (nicotinamide) and have shown its impact at the molecular, cellular, and behavioral level. If it does turn out that nicotinamide is effective in preventing alcohol-induced fetal damage in human trials, a challenge will then be to determine when intervention with the treatment is possible. In this report, the intervention was given at varying time points after a single fixed dose of ethanol. But giving any therapy during pregnancy is inherently difficult, both for the potential other effects of the treatment on the developing embryo and for the implicit difficulties in identifying alcohol use during pregnancy.

## Clinical Implications

For clinicians and clinical researchers, this new study provides insights into the roles of caspase-3 and the release of cytochrome-c in alcohol-induced damage in the developing brain, and into the use of an interventional agent that targets this damage. This study and others suggest that there is potential that a therapy may be available in the future for preventing FAS and FASD. However, there are many hurdles to clear before such a therapy is available.

For a start, it is difficult to identify the use of alcohol or other substances of abuse during pregnancy, as women themselves are unlikely to volunteer this information. And even if we can identify women who drink during pregnancy, there are many variables that could influence the success of an intervention to prevent FAS or FASD: (1) the wide range of ways in which pregnant women use alcohol (e.g., regular drinking, binge drinking, or just one-off drinking); (2) the continuously developing embryo's different vulnerabilities to alcohol-induced damage (the impact of alcohol is different during organogenesis compared with that during second or third trimesters); (3) the woman's genetic background; (4) the possibility of additive effects from other exposures (nicotine, environmental factors, other drugs); and (5) the possibility that the intervention itself turns out to be teratogenic. We still do not know how all these variables would affect the success of a therapeutic intervention given during pregnancy.

An alternative strategy would be to develop interventions that are administered once a developmental problem is identified in the child, i.e., long after the initial alcohol exposure. For example, perhaps there may be value in giving nicotinamide or other therapies to a school-aged child with learning impairment due to FAS or FASD. Ultimately, this alternative strategy may end up being a more valuable one, as it targets children with known problems and avoids the risks of teratogenicity associated with giving a drug during pregnancy.

**Box 1. Warning Labels on Alcohol**

The Omnibus Drug Initiative Act of 1988 enacted by the US Congress required that all containers of alcoholic beverages produced for domestic consumption carry the following warning labels, effective November 19, 1989: “l. According to the Surgeon General, women should not drink alcoholic beverages during pregnancy because of the risk of birth defects. 2. Consumption of alcoholic beverages impairs your ability to drive a car or operate machinery, and may cause health problems.”

## Abbreviations

FAS: fetal alcohol syndrome
FASD: fetal alcohol spectrum disorder
